# Screening for extended spectrum beta-lactamase–producing organisms in colorectal and genitourinary tract surgeries at the Lebanese American University Medical Center-Rizk Hospital

**DOI:** 10.1097/MD.0000000000047891

**Published:** 2026-02-28

**Authors:** Anna Farra, Mariana Helou, Ramzi Nakhle, Khalil Richa, Omar El Tarras, Sana Zoghbi, Ibrahim Ismail, Rola Husni

**Affiliations:** a Department of Internal Medicine, Division of Infectious Diseases, Lebanese American University, School of Medicine, Beirut, Lebanon; b Department of Internal Medicine, Division of Emergency, Lebanese American University, School of Medicine, Beirut, Lebanon; c Department of Internal Medicine, Lebanese American University, School of Medicine, Beirut, Lebanon; d Division of Infection Control, Lebanese American University Medical Center, Beirut, Lebanon; e Department of Internal Medicine, Lebanese American University, School of Medicine, Beirut, Lebanon.

**Keywords:** colonization, ESBL, infections, Lebanon, surgical

## Abstract

Recently, the global prevalence of extended spectrum beta-lactamases (ESBL)-producing Enterobacteriaceae has been increasing, with significant implications for community-acquired and healthcare-associated infections. Although antimicrobial prophylaxis is a standard practice for preventing postoperative infections, ESBL-producing bacteria are frequently not covered by current regimens. This study aimed to assess the prevalence of ESBL carriage among patients scheduled for gastrointestinal and genitourinary tract surgeries. We conducted an observational analysis at the Lebanese American University Medical Center-Rizk Hospital in Lebanon from February 4, 2013, to July 15, 2014. The study population included 126 patients, with 23.8% ESBL carriers. Higher carriage was observed with the higher age group. In the bivariate analysis, smoking and recent antibiotic use showed a significant difference for ESBL carriage, similarly for the previous admission and the length or type of hospitalization. After the multivariate analysis, only smoking status remained a significant factor. No patients developed surgical site infection. The ESBL carriage rate in our institution is high compared with international prevalence. Smoking remained the main risk factor. Despite this, none of the recruited patients who underwent gastrointestinal or genitourinary tract surgeries developed surgical site infections. Therefore, it is suggested that infections with ESBL-producing organisms can be multifactorial and not only related to colonization alone. To explore risk factors, further larger studies are warranted.

## 1. Introduction

Antibiotic resistance is one of the most serious global health issues in the 21st century. The overuse and misuse of antibiotics significantly accelerated the emergence and spread of resistant bacteria, generally affecting patient outcomes and healthcare costs. Extended spectrum beta-lactamases (ESBLs) are a group of enzymes produced by gram-negative bacteria, especially Enterobacteriaceae such as *Escherichia coli (E coli*) and *Klebsiella* species. These enzymes provide resistance against a wide range of beta-lactam antibiotics, including third- and fourth-generation cephalosporins and monobactams, but not cephamycins or carbapenems.^[1]^

Recently, the global prevalence of ESBL-producing Enterobacteriaceae has been increasing, with significant implications for community-acquired and healthcare-associated infections.^[2]^ Recent studies in Lebanon have viewed this fact with concern, as the incidence of ESBL infections has been surging, accounting for 30% of all *E coli* isolates cultured in the hospital setup.^[3]^ Colonization with ESBL-producing organisms (ESBL carriages) can lead to serious infections, which is particularly essential in surgical patients, as infection with resistant organisms still has a significant impact.^[3]^

Patients can be asymptomatic carriers of ESBL-producing bacteria, frequently harboring these organisms in the gastrointestinal (GI) tract. This colonization becomes particularly difficult during surgeries involving the lower GI or urogenital regions, where the risks of contamination and subsequent infection are increased.^[4]^ Surgical site infections (SSIs) are a significant concern, as they can lead to prolonged hospital stays, increased morbidity, and higher healthcare costs. Studies have shown that the presence of ESBL carriage doubles the risk of SSI in colonized patients.^[5]^

Although antimicrobial prophylaxis is a standard practice for preventing postoperative infections, ESBL-producing bacteria are frequently not covered by current regimens.^[6]^ This gap in coverage necessitates the consideration of targeted antimicrobial prophylaxis for patients identified as carriers of these resistant organisms. Evidence has suggested that such targeted strategies can reduce the incidence of postoperative infectious complications and lower overall healthcare costs.^[7]^

This study aimed to assess the prevalence of ESBL carriage among patients scheduled for GI and genitourinary (GU) tract surgeries at the Lebanese American University Medical Center-Rizk Hospital and evaluate the impact of ESBL carriage on postsurgical infectious complications.

## 2. Methodology

### 2.1. Study design

This study was conducted as a double-blind observational analysis at the Lebanese American University Medical Center-Rizk Hospital from February 4, 2013, to July 15, 2014. During the study, neither the patients nor the surgical teams were aware of the ESBL status of the participants, which was essential for maintaining the integrity of the findings.

### 2.2. Study population

The study population comprised patients scheduled for lower GI or GU tract surgeries. During the study period, a total of 131 patients who agreed to participate in this study were recruited. Patients were eligible for inclusion if they were aged 18 years or older and scheduled for elective lower GI or GU tract surgeries. Additionally, before study enrollment, all participants provided written informed consent.

### 2.3. Data collection and laboratory methods

To ensure consistency and reduce bias, data collection and laboratory analyses were performed in a systematic and blinded manner. The process began with screening for ESBL-producing Enterobacteriaceae, followed by comprehensive data collection through questionnaires and patient chart review, and concluded with laboratory testing for antibiotic susceptibility and ESBL detection. From each patient, 3 consecutive stool or rectal swab cultures were obtained. The first sample was collected during the preoperative period alongside other routine tests, whereas the subsequent samples were taken on the first and second postoperative days. Preoperatively, patients completed a standardized questionnaire that covered potential risk factors for ESBL carriage and postoperative infections. To maintain consistency, the same researcher conducted all interviews. Any missing data were retrieved from patient medical records.

All patients received identical preoperative prophylaxis in accordance with the hospital’s protocol based on Johns Hopkins guidelines. Patients were monitored for 2 months postoperatively through clinic visits and follow-up phone calls, with any infectious complications meticulously recorded.

Using the disk diffusion method, antimicrobial susceptibility of the isolates was evaluated, targeting 17 antibiotics, including cefotaxime, ceftriaxone, ceftazidime, aztreonam, and others. The tests were conducted on Mueller–Hinton agar using the flooding technique, and the results were interpreted following the Clinical and Laboratory Standards Institute guidelines, created in 2005. *E coli* ATCC25922 and *Klebsiella pneumoniae (K pneumoniae*) ATCC700603 served as quality control strains.

The presence of ESBL production in bacterial isolates was confirmed using the standard disk diffusion method with combined discs (ceftazidime/clavulanate and cefotaxime/clavulanate). Additionally, the *E* test method was employed using double-sided strips containing either ceftazidime or cefotaxime on one side and the same antibiotic combined with clavulanate on the other, as per Clinical and Laboratory Standards Institute recommendations.

### 2.4. Data analysis

The data analysis was performed using Statistical Package for the Social Sciences version 27 and encompassed descriptive, bivariate, and multivariate statistical methods to explore the relationships between ESBL status and various demographic, clinical, and household factors.

Initially, descriptive statistics were calculated to provide an overview of the study population. Subsequently, bivariate analyses were performed to identify potential associations between ESBL carriage status and individual predictor variables. Multivariate logistic regression was conducted to further investigate these relationships and control for potential confounding factors.

### 2.5. Ethical considerations

All participants were informed verbally about the study and provided written informed consent before inclusion. This study was conducted in accordance with the ethical standards of the institutional research committee and with the 1964 Declaration of Helsinki and its later amendments. The study was approved by the Lebanese American University Committee on Human Subjects in Research, UMCRH.AF1.12/Dec/2012.

## 3. Results

Overall, 131 patients were initially recruited, 40 and 91 of whom were female and male patients, respectively. Among the included patients, 5 received carbapenem (meropenem) as prophylaxis and were subsequently excluded.

### 3.1. ESBL-producing organism colonization (ESBL carriage) and risk factors

The study population included 126 patients, with 40 females and 86 males. Among them, 30 patients (23.8%) were ESBL carriers, with 12, 9, and 9 being 1-, 2-, and 3-sample positive, respectively.

Of the 30 ESBL carrier patients, only 1 had a specimen of *K. pneumoniae* ESBL, sensitive to ciprofloxacin (cipro) and resistant to trimethoprim/sulfamethoxazole (t/s). The remaining 29 patients (96.6%) were *E coli* ESBL-producing, 13 (45%) and 20 (69%) of whom were sensitive to cipro and t/s, respectively.

**Figure 1.**
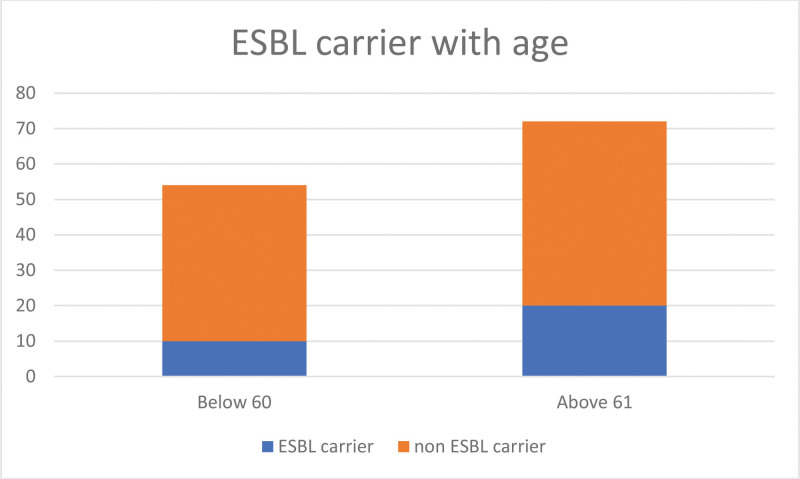
Distribution of ESBL carriers and non-ESBL carriers across 2 age groups (<60 years and >61 years). ESBL = extended spectrum beta-lactamases.

Higher carriage was observed with the higher age group. Of the patients, 72 (57%) were in the last 2 age groups (between 61 and 70 years or >71 years), and 20 (27.7%) were ESBL carriers. Moreover, 54 patients were in the lower age groups, all aged <60 years, and 10 (17%) were ESBL carriers, as shown in Figure 1.

ESBL carriage was comparable, independent of gender, with 22.5% and 25% of the female and male patients being ESBL carriers, respectively.

The carriage rate seemed similar for workers and nonworkers but higher in retirees. Of the 126 patients, 66 had worked, 12 (18.2%) of whom were ESBL carriers; additionally, 29 were retired, 11 (37.9%) of whom were ESBL carriers; and 31 were unemployed, students, or housewives, 7 (22%) of whom were ESBL carriers.

Healthcare workers had ESBL carriage rates similar to non-healthcare workers. Of the patients, 11 worked within the healthcare setting, 4 (36%) of whom were ESBL carriers.

No difference was observed in the carriage with hospital contact. Furthermore, 17 patients had a member of their family working in the medical field, 4 (23.5%) of whom were ESBL carriers. The remaining 109 did not work in the medical field, 26 (23.8%) of whom were ESBL carriers.

Regarding the area of primary residency, of the patients, 39 lived in Beirut, 12 (30.8%) of whom were ESBL carriers; 58 lived in Mount Lebanon, 10 (17.2%) of whom were ESBL carriers; 15 lived in the South, 3 (20%) of whom were ESBL carriers; and 14 lived in other areas, 5 (25.7%) of whom were ESBL carriers.

One patient who lived in Africa was not an ESBL carrier, whereas 1 patient who lived in Iraq was an ESBL carrier.

Additionally, 5 patients were bedridden at home with care, 2 of whom were ESBL carriers.

Regarding the patients with planned surgeries, of the 67 with planned GI tract surgeries, 19 (28%) were ESBL carriers, whereas of the 64 patients with planned GU tract surgeries, 13 (20% carriers before surgery) were ESBL carriers.

The duration of most surgeries ranged between 1 and 2 hours. However, 16 surgeries lasted for >4 hours.

Only 1 patient developed a complication during surgery (bleeding) and was not an ESBL carrier.

Patients were asked about previous surgeries in the last year. Of the patients, 31 had previous surgeries, 12 (38.7%) of whom were ESBL carriers; additionally, 95 did not undergo surgeries, 18 (18.9%) of whom were ESBL carriers, indicating that the group that had previous surgeries had more ESBL carriers. Cystoscopy was the most common type of previous surgery, accounting for 31 patients, 16 (37.5%) of whom were ESBL carriers.

Thirty-seven patients responded being sick in the last 3 months, 12 (32.4%) of whom were ESBL carriers. Moreover, 89 patients responded being not sick, 18 (20.2%) of whom were ESBL carriers. The most common sickness was respiratory infection (14 patients, 5 of whom were ESBL carriers), followed by dental diseases (6 patients, 3 of whom were ESBL carriers).

Forty-four patients reported the use of antibiotics in the last 3 months, 16 (3%) of whom were ESBL carriers. Moreover, 81 denied any antibiotic use, 14 of whom were ESBL carriers.

During the past year, 51 patients were hospitalized, 19 (37.2%) of whom were ESBL carriers, whereas of the 75 patients, who did not require hospitalization, 11 (14.7%) were ESBL carriers.

Cystoscopy (15 patients) was the most common reason for hospitalization.

On questionnaire day, patients were asked about antimicrobial use or any other treatment. Among the patients who were ESBL carriers, 1 received quinolones as prophylaxis and 2 received amoxicillin–clavulanate.

### 3.2. Bivariate analysis

In the bivariate analysis, smoking and recent antibiotic use showed a significant difference for ESBL carriage (*P* = .037 and .028, respectively). A significant difference for ESBL carriage was also noted in previous admission and the length or type of hospitalization (Table 1).

**Table 1:** Bivariate analysis of ESBL versus non-ESBL across demographic, household factors, clinical, surgery-related, and post-OP factors.

Variable	Test statistic	*P*-value
Demographic factors		
Age	1.073	.283
Gender	0.166	.734
Occupation status	4.106	.250
Type of occupation	5.054	.282
Residence type	–	–
Mohafaza	10.213	.116
Household factors		
Household member numbers	−1.668	.095
Household members in medical field	0.009	.926
Household members that are bedridden	0.683	.409
Household members with underlying conditions		
Diabetes	3.960	.055
Cancer	0.055	.814
Organ transplant	–	–
UTI	0.415	.519
Rheumatologic	0.132	.716
Defect immunity	0.719	.396
Household members' surgery in the past year	0.431	.511
Household members on antibiotics in the past 3 mo	0.554	.758
Household members' illness in the past 3 mo	0.025	.873
Household members' recent hospitalization	0.487	.485
Household members' hospitalization length of stay	−0.383	.703
Household members' antibiotic use	0.554	.758
Medical and clinical factors		
BMI	−1.109	.267
Smoking status	6.574	.037*
Malignancy	6.914	.141
Medication		
Quinolones	0.719	.396
Co-amoxiclav	6.283	.012
Cephalosporin	–	–
PPI	0.392	.531
Probiotics	–	–
Comorbidities		
Diabetes	0.043	.836
Cancer	2.396	.122
Organ transplant	–	–
UTI	1.855	.173
Rheumatologic	0.326	.538
Defect immunity	0.719	.396
Previous surgery	3.404	.065
Illness in the past 3 mo	1.210	.271
Antibiotic in the past 3 mo	7.169	.028*
Type of antibiotic in the past 3 mo	7.603	.369
Previous hospitalization	5.759	.016*
Hospitalization length of stay	2.316	.021*
Type of hospitalization	9.470	.024*
Urinary catheter in the past 3 mo	0.616	.433
Previous MDRO	0.541	.763
Type of MDRO	0.55	.814
Surgery-related factors		
Type of current surgery	1.148	.284
Uncontrolled blood sugar perioperative	2.205	.335
Antibiotic prophylaxis	0.345	.842
Surgical complication	0.326	.568
Pre-OP antibiotic	0.214	.644
Pre-OP antibiotics duration	0.173	.219
Post-Op factors		
Post-Op antibiotics	0.004	.948
Post-Op antibiotics duration	0.848	.396

–: no statistics are computed because the variable is a constant.

BMI = body mass index, ESBL = extended spectrum beta-lactamases, MDRO = multidrug-resistant organism, PPI = proton pump inhibitors, UTI = urinary tract infection.

* *P* < .05 indicates a statistically significant association at the 5% level.

### 3.3. Use of antibiotics

The choice of antibiotic for prophylaxis was not performed as per protocol in 46 patients.

The antibiotic prescribed as prophylaxis against ESBL-producing organisms was administered to patients who were not ESBL carriers, similar to those who were ESBL carriers.

Most patients who were ESBL carriers received antibiotics that were not active against ESBL-producing organisms.

### 3.4. Multivariate analysis

Multivariate analysis was performed on a simple model and revealed that when all the factors were added together, the factors that showed a significant difference in ESBL carriage were no longer significant. Only smoking status remained a significant factor (Table 2).

**Table 2:** Complex model of multivariate logistic regression for predictors of ESBL status.

Predictor	*B*	S.E.	Wald	Sig. (*P*-value)	Exp (*B*) (odds ratio)
Medication_coamoxiclav (1)	−45.665	26271.482	0.000	.999	0.000
Antibiotics past 3 mo			6.413	.040	
Antibiotics past 3 mo (1)	19.002	40193.170	0.000	1.000	178884315.975
Antibiotics past 3 mo (2)	20.596	40193.170	0.000	1.000	880526647.760
Hospitalized past year (1)	20.798	25374.749	0.000	.999	1077905210.830
Hospitalized_length of stay			2.475	.649	
Hospitalized_LOS (1)	−23.677	25374.749	0.000	.999	0.000
Hospitalized_LOS (2)	−1.923	2.355	0.666	.414	0.146
Hospitalized_LOS (3)	−3.413	2.567	1.768	.184	0.033
Hospitalized_LOS (4)	−23.338	9078.067	0.000	.998	0.000
Hospitalized_type			0.000	1.000	
Hospitalized_type (1)	−44.305	34463.439	0.000	.999	0.000
Hospitalized_type (2)	−44.268	34463.439	0.000	.999	0.000
Hospitalized_type (3)	−83.077	68079.429	0.000	.999	0.000
Comorbidities_diabetes (1)	1.227	0.905	1.837	.175	3.410
Comorbidities_cancer (1)	−0.787	0.695	1.285	.257	0.455
Comorbidities_UTI (1)	−0.952	1.012	0.885	.347	0.386
Comorbidities_rheumatologic (1)	19.367	40192.933	0.000	1.000	257686553.090
Comorbidities_defectimmune (1)	−1.635	1.794	0.830	.362	0.195
MDRO_history			0.000	1.000	
MDRO_history (1)	20.725	40193.056	0.000	1.000	1002143406.855
MDRO_history (2)	−1.990	42317.718	0.000	1.000	0.137
Household members_medical field (1)	0.532	0.809	0.432	.511	1.702
Pre-OP_antibiotic (1)	0.288	0.919	0.098	.754	1.334
Post-OP_antibiotics (1)	−0.463	0.845	0.300	.584	0.630
BMI	0.034	0.044	0.601	.438	1.035
Smoking status			5.812	.055	
Smoking status (1)	−39.737	13592.031	0.000	.998	0.000
Smoking status (2)	−41.315	13592.031	0.000	.998	0.000
Gender (1)	−0.577	0.652	0.782	.376	0.562
Age			4.186	.651	
Age (1)	44.151	41205.413	0.000	.999	14938930794302466000.000
Age (2)	−56.163	16943.296	0.000	.997	0.000
Age (3)	1.395	1.109	1.583	.208	4.034
Age (4)	1.370	1.024	1.790	.181	3.934
Age (5)	−1.391	1.357	1.049	.306	0.249
Age (6)	0.527	0.837	0.396	.529	1.693

Variables entered into model: medications_coamoxiclav, antibiotics past 3 mo, hospitalization past yr, hospitalization_LOS, hospitalization_type, comorbidities, diabetes, comorbidities_cancer, comorbidities_UTI, comorbidities_rheumatologic, comorbidities_defectimmunity, MDRO_history, household member_medical field, BMI, smoking status, gender, age.

BMI = body mass index, ESBL = extended spectrum beta-lactamases, LOS = hospitalization length of stay, MDRO = multidrug-resistant organism, UTI = urinary tract infection.

No patients developed SSI.

## 4. Discussion

This is a prospective study mainly aimed to identify the prevalence of ESBL carriage in patients planned for colorectal and GU tract surgeries and to determine the impact of ESBL carriage and the type of prophylactic antibiotherapy on postsurgical infectious complications.

In our study, the ESBL carriage rate of 23.8% was comparable with the prevalence of ESBL *E coli* intestinal carriage in the Eastern Mediterranean of 20.6% in a meta-analysis published in 2020.^[8]^ The same study determined a pooled prevalence of ESBL *E coli* intestinal carriage in the community of 16.5%, with the highest carriage rate in Southeast Asia of 27% and the lowest in Europe (6%) and the USA (3.5%).^[8]^ This meta-analysis showed a prevalence rate in Lebanon of 38.5%, which is even higher than the rate reported in the present study. This finding may be explained by the small number of patients recruited in the meta-analysis and the fact that our study is a single-center study. The majority of our patients lived in the Mount Lebanon area, which is less condensed than Beirut and has the lowest rate of ESBL carriage at 17.2%.

Our findings highlighted a higher ESBL carriage rate in the older age group (>61 years). This observation aligns with the international ESBL carriage prevalence. A study conducted in Canada evaluated the rates of ESBL-producing Enterobacterales isolated from urine cultures during the coronavirus disease 2019 pandemic and reported that patients aged >60 years had significantly higher ESBL *E coli* and *K pneumoniae* rates than the younger age groups.^[9]^ This finding can be attributed to possible higher antibiotic use and healthcare exposure in older adults, potentially leading to a higher carriage state. A study conducted in Sweden showed similar high ESBL rates among older adult patients living in nursing homes and older adults living in their homes.^[10]^ Similarly, in our study, higher rates were observed in bedridden patients, which may be due to age, comorbidities, and previous antibiotic use.

Notably, the ESBL carriage rate in our population is similar between working and nonworking individuals (students and housewives) but higher in retired individuals. The high ESBL carriage rate in retired individuals is believed to be attributed to higher age.

A recently published study from Thailand^[9]^ did not find a significant difference in ESBL carriage rates between females (22.5%) and males (25%). However, Hasan et al reported that ESBL-producing *E coli* and *K Pneumoniae* rates were higher in the male group.^[11]^ Differences could be attributed to our small population size and other confounding factors.

In our study, recent antibiotherapy is not a risk factor for ESBL carriage, which is likely due to the short time window between the initiation of antibiotic use and the time of culture.^[12]^ To better evaluate the potential correlation, a larger study with more detailed analyses is needed.

Several studies have evaluated proton pump inhibitor (PPI) use as a risk factor for Enterobacteriaceae infection. The relationship between PPI use and rectal carriage of ESBL Enterobacteriaceae at hospital admission was examined, and it was found that PPI use was independently associated with carriage. Compared with 2.9% of non-PPI users, 8.5% of PPI users were rectal carriers of ESBL-Enterobacteriaceae. In our study, we noted that that 20% of PPI users were ESBL carriers compared with 24% of non-PPI users. This finding is most probably attributed to our high ESBL carriage rate.

In this study, no significant association was observed between ESBL carriage and various factors, including having a family member working in the medical field or prior hospital contact. However, hospital admission in the last 6 months is known to be a risk factor for ESBL carriage.^[13]^ Nevertheless, from our population, 37.2% of the patients who were hospitalized during the last year were ESBL carriers, which was higher than that of the nonhospitalized group.

Patients with comorbidities, including diabetes, malignancy, immunodeficiency, and recurrent urinary tract infections, had higher ESBL carriage rates. Patients with diabetes mellitus are more vulnerable to infections owing to alterations in host defenses and compromised immunological function. They are more likely to be exposed to ESBL-producing bacteria as they frequently require healthcare visits and antibiotic use.^[14]^ Chemotherapy, radiation therapy, and surgery are frequently administered to patients with cancer, which may compromise their immune system and make them more vulnerable to colonization, including those caused by ESBL-producing bacteria.^[15]^

In our study, smoking was the only factor that remained significant for ESBL carriage following multivariate analysis. Nicotine use was previously cited as a risk factor for fecal colonization with ESBL-producing Enterobacterales.^[16]^ Moreover, smoking has been proven to change the microbiome, which could be a contributing factor.^[17]^ Additionally, a vegetarian diet protects against ESBL carriage. Meat in the diet is a risk factor. In Lebanon, raw meat is a common diet trend, and animals receive antibiotics as a growth factor, which could select out for ESBL-producing organisms.^[16]^

Studies have shown that enteric colonization with ESBL-producing Enterobacteriaceae increased the risk of SSI in GI tract surgeries despite standard prophylaxis regimens. The probability of SSI, specifically deep SSI, was more than doubled in multivariable analysis by ESBL-PE carriage. Among noncarriers and carriers, 1.6% and 7.2%, respectively, developed SSI due to ESBL-PE.^[18]^ Depending on the study and patient population, the risk of SSI among ESBL-colonized individuals undergoing GI tract surgeries, including colorectal procedures, may vary from 10% to ≥30%.^[18]^

Although the percentages can vary, GU tract surgeries, including those affecting the urinary system, may also increase SSI risk in patients with ESBL colonization.^[19]^ In our study, no patients developed SSI. This finding can be explained by the fact that other factors can affect SSI risk in patients with ESBL colonization, including the presence of comorbidities, invasive device use, length of surgery, infection control protocols, and surgical complications.^[20]^ In our recruited patients, most surgeries were uncomplicated and had short durations. Additionally, all surgeries were performed under strict infection control protocols, which could explain why none of them developed infections.

## 5. Conclusion

The ESBL carriage rate in Lebanon, particularly in our institution, is high compared with international prevalence. In our study, the ESBL carriage rate was higher in older adults and patients who were hospitalized in the previous year. Comorbidity was frequently associated with frequent healthcare facility visits, broad-spectrum antibiotic use, and invasive procedures, all of which increased the likelihood of ESBL colonization. We neither observed a gender-based difference, a higher carriage in family members of healthcare professionals, nor a correlation with recent antibiotherapy. Smoking remained the main risk factor. Patients with ESBL colonization were generally associated with an increased risk of SSIs compared with those without ESBL colonization. However, despite the high ESBL carriage rate in our population, none of the recruited patients who underwent GI or GU tract surgeries developed SSI. Therefore, it is suggested that infections with ESBL-producing organisms can be multifactorial and not only related to colonization alone. None of our patients had a long or complicated course, which may have protected them against infection. To explore risk factors, further larger studies are warranted.

## Author contributions

**Conceptualization:** Anna Farra, Mariana Helou, Ramzi Nakhle, Khalil Richa, Omar El Tarras, Sana Zoghbi, Rola Husni.

**Data curation:** Anna Farra, Mariana Helou, Ramzi Nakhle, Khalil Richa, Omar El Tarras, Sana Zoghbi, Rola Husni.

**Formal analysis:** Anna Farra, Mariana Helou, Ibrahim Ismail, Rola Husni.

**Investigation:** Anna Farra, Mariana Helou, Rola Husni.

**Methodology:** Anna Farra, Mariana Helou, Rola Husni.

**Project administration:** Anna Farra, Mariana Helou, Rola Husni.

**Resources:** Anna Farra, Mariana Helou, Rola Husni.

**Supervision:** Mariana Helou, Rola Husni.

**Software:** Ibrahim Ismail.
